# Feature selection and nearest centroid classification for protein mass spectrometry

**DOI:** 10.1186/1471-2105-6-68

**Published:** 2005-03-23

**Authors:** Ilya Levner

**Affiliations:** 1 Department of Computing Science, University of Alberta, Canada

## Abstract

**Background:**

The use of mass spectrometry as a proteomics tool is poised to revolutionize early disease diagnosis and biomarker identification. Unfortunately, before standard supervised classification algorithms can be employed, the "curse of dimensionality" needs to be solved. Due to the sheer amount of information contained within the mass spectra, most standard machine learning techniques cannot be directly applied. Instead, feature selection techniques are used to first reduce the dimensionality of the input space and thus enable the subsequent use of classification algorithms. This paper examines feature selection techniques for proteomic mass spectrometry.

**Results:**

This study examines the performance of the nearest centroid classifier coupled with the following feature selection algorithms. Student-t test, Kolmogorov-Smirnov test, and the P-test are univariate statistics used for filter-based feature ranking. From the wrapper approaches we tested sequential forward selection and a modified version of sequential backward selection. Embedded approaches included shrunken nearest centroid and a novel version of boosting based feature selection we developed. In addition, we tested several dimensionality reduction approaches, namely principal component analysis and principal component analysis coupled with linear discriminant analysis. To fairly assess each algorithm, evaluation was done using stratified cross validation with an internal leave-one-out cross-validation loop for automated feature selection. Comprehensive experiments, conducted on five popular cancer data sets, revealed that the less advocated sequential forward selection and boosted feature selection algorithms produce the most consistent results across all data sets. In contrast, the state-of-the-art performance reported on isolated data sets for several of the studied algorithms, does not hold across all data sets.

**Conclusion:**

This study tested a number of popular feature selection methods using the nearest centroid classifier and found that several reportedly state-of-the-art algorithms in fact perform rather poorly when tested via stratified cross-validation. The revealed inconsistencies provide clear evidence that algorithm evaluation should be performed on several data sets using a consistent (i.e., non-randomized, stratified) cross-validation procedure in order for the conclusions to be statistically sound.

## Background

Advances in protein mass spectrometry have have recently shown great potential for high-throughput disease classification and biomarker identification. In turn, fast and accurate detection of diseases, such as early cancer detection, can revolutionize the field of medical diagnosis. Typically, serum samples are analyzed by a mass spectrometer, producing a high dimensional abundance histogram. Next, informative features are extracted from the high dimensional data and are presented to a classifier. In turn, the classifier outputs a decision about the status of the patient with respect to a particular disease (e.g., healthy or diseased). Recently, numerous feature selection and classification techniques have been shown to perform well on several isolated data sets. However, current literature does not contain rigorous comparative studies analyzing the merits of individual feature selection and classification algorithms across several data sets. This paper analyzes several state-of-the-art feature selection methods coupled with a very fast nearest centroid classifier. In addition, we present a novel combination of boosted feature extraction coupled with the nearest centroid classifier, which consistently outperforms all other algorithms tested in terms of classification accuracy.

### Mass spectrometry analysis

Discovered by Sir J.J. Thomson in the early part of the 20^*th *^century Mass spectrometry (MS) is a technique for 'weighting' individual molecules, fragments of molecules or individual atoms that have been ionized. In a vacuum environment an ion source vaporizes and charges the sample matter, which is then deflected into a magnetic or electric field. The mass spectrometer then measures the molecular masses along with abundances and masses of fragments that are produced as a result of molecular breakdown. The fundamental measurement unit of the MS is the mass-to-change ratio (M/Z). For proteomic applications, Daltons (Da) are used to measure mass, while the electric potential of a single electron is the measurement unit for charge (z). The spectrum is a graph of ion intensity as a function of mass-to-charge ratio and is often depicted as a histogram.

#### Time-of-Flight (TOF)

In time-of-flight (TOF) instruments, positive ions are produced by periodic bombardment of the sample with brief pulses of either electrons, secondary ions, or laser-generated photons. The ions produced by the laser are then accelerated by an electric field pulse and passed into a field-free drift tube. Ideally, all ions entering the tube will have the same kinetic energies, and their velocities must therefore vary inversely with their masses, with lighter particles arriving at the detector earlier than the heavier ones. The ions therefore drift through a field-free path and are separated in space and time-of-flight [5].

#### Matrix assisted laser desorption/ionization (MALDI)

By incorporating the (bio)molecules in a large excess of matrix molecules, strong intermolecular forces are reduced. The matrix molecules absorb the energy from the laser light and transfer it into excitation energy of the solid system. The effect is an instantaneous phase transition of small molecular layers of the sample into a gaseous state. Thus solid (and liquid) material can be easily analyzed by TOF MS.

#### Surface-enhanced laser desorption/ionization (SELDI)

This method uses protein chip arrays with different selective surfaces such as cation or anion exchange surfaces, hydrophobic surfaces and metal binding surfaces. Biofluids such as cell lysate, plasma or urine are applied onto the selective surface and, after washing, a subset of proteins is specifically bound. The chip is then analyzed in a (MALDI) TOF-MS which generates a protein spectrum of the different molecular masses present on the protein chip. This technology is therefore highly suited for research into molecular mechanisms of disease and biomarker identification.

### Related research

Mass Spectrometry (MS) based pattern recognition is rapidly becoming a broad and fruitful research field. This section, provides details on current state of research within the field of early cancer diagnosis based on proteomic pattern recognition.

#### Ovarian cancer studies

In [19], genetic algorithms together with self-organizing maps were used to distinguish between healthy women and those afflicted with ovarian cancer. Although cross-validation studies were not conducted, the approach was able to correctly classify all cancer stricken patients and 95% of healthy women, on a single test set.

Using the same data sets in [17], the researchers employed Principle Component Analysis (PCA) [14] for dimensionality reduction and Linear Discriminant Analysis (LDA) [8] coupled with a nearest centroid classifier [18] for classification. For each of the train/test data splits, 1000 cross-validation runs with re-sampling were conducted. When training sets were larger than 75% of the total sample size, perfect (100%) accuracy was achieved on the OC-WCX2b data set. Using only 50% of data for training, the performance dropped by 0.01%. Unfortunately, the probabilistic approach used in this study can leave some samples unclassified. For the OC-H4 data set, the system had a 92.45% sensitivity and 91.95% specificity when 75% of the data was used for training. However, only 98.60% of the data samples were classified. Similarly, for the OC-WCX2a data set 97.34% sensitivity and 96.99% specificity was attained on 99.92% of the test data, when 75/25 train/test split was used.

In [30], the researchers compared two feature extraction algorithms together with several classification approaches on a MALDI TOF acquired data. The T-statistic, also known as the student-t test [21], was used to rank features in terms of their relevance. Then two feature subsets were greedily selected (respectively having 15 and 25 features each). Support vector machines (SVM), random forests, linear/quadratic discriminant analysis (LDA/QDA), k-nearest neighbors, and bagged/boosted decision trees were subsequently used to classify the data. In addition, random forests were also used to select relevant features with previously mentioned algorithms used for classification. Again 15 and 25 feature sets were selected and classification algorithms applied. When the T-statistic was used as a feature extraction technique, SVM, LDA and random forests classifiers obtained the top three results (with accuracy in the vicinity of 85%). On the other hand, classification improved to approximately 92% when random forests were used as both feature extractors and classifiers. Similar performance was also achieved using the the nearest-neighbor algorithm, a close relative of the nearest centroid algorithm [28] we will be using in this study. While the results appear promising, the authors provide little motivation as to why 15 and 25 feature sets were selected. Other that the fact that LDA and QDA need the number of features to be less than the number of samples, the actual size of the selected feature set seems to be an arbitrary choice. In practice, determining the size of the feature set is an added burden, placed on the software developer and, ideally, should be eliminated. Furthermore, testing several feature sets of various sizes and selecting the set with the best performance can lead to overfitting. With that in mind we propose to automatically select features and the size of the feature set using an internal leave-one-out cross-validation procedure (LOOCV) discussed in the following sections.

Using the same MALDI TOF data set as in [30], researchers in [26] applied the nearest shrunken centroid approach to classify the MS samples. Using only seven features their method achieved a classification error rate of approximately 23%. More recently, in [12], both the GA approach and the nearest shrunken centroid approach have been found inferior to the boosting based feature selection approach. Further investigation, in [16], confirmed the poor performance of the nearest shrunken centroid on the ovarian cancer (OC-H4) and the prostate cancer (PC-H4) data sets.

### Prostate cancer studies

In [1], the researchers used a decision tree algorithm to differentiate between healthy individuals and those with prostate cancer. This study used the SELDI TOF MS to acquire the mass spectra which corresponds to our PC-IMAC-Cu data set. In order to select relevant features, the area under the Receiver Operating Characteristics (ROC) curves was used to identify informative peaks which were subsequently used by the decision tree classification algorithm. The researchers did not perform cross-validation, but on a single test set the classifier achieved an 81% sensitivity and a 97% specificity, yielding a balanced accuracy (BACC) of 89%.

In [22], the performance was improved on the PC-IMAC-Cu data set by the use of boosting. As is [1], the area under the curve (AUC) criteria was used to identify relevant features. For subsequent feature selection and classification, the researchers used decision stumps together with AdaBoost and its variant, Boosted Decision Stump Feature Selection (BDSFS) method. A key difference between the two methods is that BDSFS selects features without replacement, whereas boosted decision stumps (BDS) allows for selection of the same feature multiple times. The BDS algorithm achieved perfect accuracy on the single test set for the prostate cancer data set. However, a randomized 10-fold cross-validation procedure yielded an average sensitivity of 98.5% and an average specificity of 97.9%, for an overall BACC of 98%. For the BDSFS, the results were considerably worse, with a sensitivity of 91.1% and a specificity of 94.3%. The BDS algorithm used all 124 features selected by the AUC, and required 500 rounds of boosting. On the other hand, the BDSFS algorithm used just 21 features which were easily interpretable. The researchers informally report that other classifiers had similar classification accuracies but were more difficult to interpret. Although, this is the highest reported accuracy on this data set, the BDS algorithm [9] required over 500 rounds of boosting which complicates the identification of key relevant features necessary to differentiate heathy individuals from those afflicted with prostate cancer.

In [29], the same PC-IMAC-Cu data set was analyzed using several classifiers. Using a filter-based ANOVA F-statistic to rank the preselected peaks, relevant features were selected in sets of increasing size. Classification was performed with k-nearest-neighbors (kNN), linear/quadratic discriminants (LDA/QDA), and suport vector machines (SVM) using 100-fold randomized cross-validation strategy. Linear SVM achieved the best accuracy of 91% using just eight peaks.

In [17], the researchers again used PCA for dimensionality reduction and LDA for classification. The PC-IMAC-Cu data set was obtained from the authors of [1] and, in the same fashion as with the ovarian cancer set, the researchers conducted a detailed study using various train/test set sizes. For each train/test data split, 1000 cross-validation runs (with re-sampling) were conducted. When training sets were larger than 75% of the total sample size, average accuracy of 88% was achieved (88.46% sensitivity and 88.98% specificity). Using only 50% of data for training, the performance dropped to 86%. In comparison to ovarian cancer sets, the lower accuracy suggests that this data set is much more difficult to classify correctly using the PCA/LDA algorithm.

In [20,31], researchers used Genetic Algorithms (GA's) for feature selection and Self Organizing Maps (SOM's) for classification of prostate cancer (data set PC-H4 in our study). This approach achieved a specificity of 95% and a sensitivity of 71%, for an average accuracy of 83%. Although cross-validation was carried out, the results were not presented.

In [6], the aforementioned studies on prostate cancer raised the following question: Why do the features and classification performance vary so drastically across studies? The results indicate that different SELDI-TOF approaches combined with different machine learning techniques for pattern recognition produce highly variable results in terms of relevant features and classification accuracy. Furthermore, such results also indicate that the MS spectra contains a large number of features relevant to the task of discriminating heathy individuals from those afflicted with cancer. An alternative explanation, found in [2], seems to suggest chemical/electronic noise and/or bias introduced during the acquisition of the MS spectra. This further motivates the need for comparative studies done on a regular basis using several mass spectrometry techniques in conjunction with a number of machine learning approaches done on several data sets.

### Data sets

For this study five data sets were acquired. Each sample in each data set is represented as a vector of real valued features forming the spectra. Each feature in turn represents the quantity (parts per million) of ions with a specific m/z ratio. In essence, each sample spectrum is a histogram describing the composition of the sample bio-fluid or tissue sample. Each data set is named based on the type of disease tested, **OC **for Ovarian Cancer and **PC **for Prostate Cancer, as well as the type of SELDI affinity chip used to produce the mass spectra. This naming scheme was adopted from [17]. The following data sets were used for this study:

#### OC-H4

This ovarian cancer set was obtained using the H4 protein chip from Ciphergen. It contains 100 diseased and 100 healthy samples which were manually prepared. Each spectra contains 15,156 features (M/Z values) in this data set.

#### OC-WCX2a

This ovarian cancer set obtained using the WCX2 protein chip. It contains the same 100 diseased and 100 healthy samples as the OC-H4 data set which were re-precessed using the WCX2 protein chip. For this data set the samples were also processed by hand. Each spectra contains 15,156 features (M/Z values) in this data set.

#### OC-WCX2b

This ovarian cancer set was also obtained using the WCX2 protein chip. However, a robotic instrument replaced the manual chip preparation for this data set. This data set contains 92 healthy and 162 diseased samples, all different from the two previous data sets. Each spectra contains 15,156 features (M/Z values) in this data set.

#### PC-H4

The spectra were collected using the H4 protein chip, which was prepared by hand. There are 322 total samples: 190 samples with benign prostate hyperplasia with PSA levels greater than 4, 63 samples with no evidence of disease and PSA level less than 1, 26 samples with prostate cancer with PSA levels 4 through 10, and 43 samples with prostate cancer with PSA levels greater than 10. Each sample is again a histogram composed of 15,156 features. For this set we combined samples with benign prostate hyperplasia and those with no evidence of disease into the healthy class. The rest of the samples formed the diseased class.

#### PC-IMAC-Cu

The spectra were collected using the IMAC-Cu metal binding chip, and were prepared by hand. There are 324 total samples: 167 samples with prostate cancer, 77 with benign prostate hyperplasia and 82 samples with no evidence of disease. Each sample is composed of 16,382 features. For this set we also combined samples with benign prostate hyperplasia and those with no evidence of disease into the healthy class. The rest of the samples formed the diseased class.

## Results

This section presents the evaluated feature selection algorithms in conjunction with the base classification technique. In addition the empirical evaluation results are presented.

### Centroid classification method

A fast and simple algorithm for classification is the centroid method [10,18]. This algorithm assumes that the target classes correspond to individual (single) clusters and uses the cluster means (or centroids) to determine the class of a new sample point. A prototype pattern for class *C*_*j *_is defined as the arithmetic mean:



where ***x***_*i*_'s are the training samples labeled as class *C*_*j*_. Recall that the training sample is a MS spectra represented as a multi-dimensional vector (denoted in bold). In a similar fashion, we can obtain a prototypical vector for all the other classes. During classification, the class label of an unknown sample *x *is determined as:



where *d*(***x*, *y***) is a distance function or:



where *s*(***x*, *y***) is a similarity metric. This simple classifier will form the basis of our studies. It works with any number of features and its run-time complexity is proportional to the number of features and the complexity of the distance or similarity metric used. Preliminary experiments in [15], were conducted to establish which similarity/distance metric is most appropriate for the centroid classification algorithm, and the L1 distance metric was selected. Defined by:

*L*_1 _(***x*, *μ***) = || ***x *- *μ***||_1 _    (1)

with ||*y*||_1 _=  |*y*(*i*)|, and *y*(*i*) being the value of the *i*^*th *^feature. The value *L*1(***x*, *μ***) has a linear cost in the number of features. In this study, data sets contain two classes and hence the number of calls to the distance metric is also two. Therefore, the centroid classifier, at run-time, is linear in the number of features. During training, two prototypes are computed and the cost of computing each prototype is *O*(*mN*), where *N *is the number of features and *m *is the number of training samples which belong to a given class. Note that *m *only varies between data sets and not during training or feature selection processes. Thus, we can view *m *as a constant and conclude that the centroid classifier has *O*(*N*)cost in the training phase.

### Nearest shrunken centroid

A special purpose feature selection algorithm for the nearest centroid algorithm was developed by Tibshirani et al. and presented in [10,25,26]. The algorithm, related to the lasso method, tries to shrink the class prototypes () towards the overall mean:



Briefly, the algorithm calculates:



where , ***s ***is a vector of pooled within class variances for each feature and division is done component wise. We can now view the class centroid as:



where denotes component wise multiplication. By decreasing ***d***_***j ***_we can move the class centroid towards the overall centroid. When a component of the class centroid is equal to the corresponding component of the overall mean for all classes, the feature no longer plays a part in classification and is effectively removed. Hence, as ***d***_***j ***_shrinks progressively more features are removed. To decrease ***d***_***j ***_soft thresholding is used to produce  with:



Where *d*_*j*_(*i*) is the *i*^*th*^component of the vector ***d***_***j***_. The shrunken centroid is then computed by replacing ***d***_***j ***_with  in equation 4. In our experiments we used 20 different values for *δ*, NSC(20), {0.5, 1, 1.5, ..., 10}.

We also tried 200 different values for *δ *also in the range (0, 10] in increments of 0.05, but attained the same BACC score while incurring ten times the computational cost (results not shown).

### Filter-based feature selection

Filter methods attempt to select features based on simple auxiliary criteria, such as feature correlation, to remove redundant features. In order to be tractable, such approaches decouple the feature selection process from the performance component, but may ultimately select irrelevant features as a result. In general, filter-based methods are designed for a specific type of feature. Since the mass spectra is composed of continuous features, we use univariate statistical tests. Instead of selecting features by invoking a classifier as in wrapper-based approaches, univariate statistics simply rank individual features. The student-t test (T-test), the Kolmogorov-Smirnov test (KS-test) [21] and the P-test [11] algorithms are the commonly used statistics. These 'goodness-of-fit' tests compare feature values of samples belonging to class 1 to feature values of samples belonging to class 2. The goal is to determine if the feature values for class 1 come from a different distribution than those for class 2. The key difference between these tests are the assumptions they make. The T-test assumes that both distributions have identical variance, and makes no assumptions as to whether the two distributions are discrete or continuous. On the other hand, the KS-test assumes that the two distributions are continuous, but makes no other assumptions.

In the case of the T-test, the null hypothesis is *μ*_1 _= *μ*_2_, indicating that the mean of feature values for class 1 is the same as the mean of the feature values for class 2. In the case of the KS-test, the null hypothesis is *cdf*(1) = *cdf*(2), meaning that feature values from both classes have an identical cumulative distribution. Both tests determine if the observed differences are statistically significant and return a score representing the probability that the null hypothesis is true. Thus, features can be ranked using either of these statistics according to the significance score of each feature. In addition to the T-test and KS-test, we also use a simpler feature ranking criteria called the P-test and denoted as:



where *σ*_*i *_is the standard deviation for class *i*. This can be seen as a simplified version of the student-t score that ignores sample size and ranks features solely on the basis of their mean and standard deviation. Both the benefits and drawbacks of these statistical tests stem from the assumption that the features are independent. On one hand, the independence assumption makes these algorithms computationally efficient. On the other hand, the independence assumption clearly may not hold for all data sets, thereby producing suboptimal feature rankings.

In [30], the researchers used the T-test to rank each feature but chose to test classification algorithms with 15 and 25 top-ranked features, without any apparent justification. The apparent focus of their research is on comparing classifiers rather than the two feature extraction methods (T-test and random forests). In contrast, we show that feature ranking coupled with greedy forward selection using internal leave-one-out cross-validation (LOOCV) can **automatically **find a feature subset of an arbitrary size that improves performance with respect to using the centroid algorithm without any feature selection.

### Wrapper-based feature selection

Wrapper Methods attempt to evaluate feature relevance *within the context *of a given task and avoid intractability by using greedy/heuristic search methods. In other words, the number of possible subsets is greatly restricted by the greedy selection procedure, and each candidate feature subset is evaluated using the actual performance element (i.e., training a classifier/regressor using a subset of features). Thus far, a variety of greedy algorithms have been proposed to select feature sets sequentially. Sequential Forward (respectively Backward) selection (SFS and SBS) methods start from an empty (respectively full) set of features and at each step add (respectively remove) a single feature which produces the greatest increase in performance. The SFS technique, as described, is easily applicable to the MS data. On the other hand, the SBS algorithm, much like a full search over all subsets, is still computationally intractable. Our informal estimates revealed that a naive application of the SBS algorithm to all five data sets, used in this study, would take approximately 100 years to complete on the hardware platform available to us. Thus, in order to make SBS tractable, we implemented several heuristics. First, rather than searching through all features within the active set, and removing a feature that produces the *greatest *improvement in performance, we stop at the first feature whose removal does not degrade the overall performance as determined by the internal LOOCV approach. Now that each loop of SBS terminates at the first candidate feature, we can re-order the features based on the probability of each feature being irrelevant and/or redundant. To do so we use the KS-test to rank and re-order all features. Thus, the SBS search starts by first testing a feature deemed most likely to be irrelevant by the KS-test. The second heuristic added to the SBS algorithm involves recording the stoping position of the last iteration. In the standard SBS, each iteration of the algorithm tests all features in the active set. However, since the previously added heuristic lets SBS terminate the innermost loop at the first feature deemed unnecessary, re-testing previously examined features has less utility than looking at the uninspected features. Hence, rather than re-starting the search from the beginning, each iteration of the modified SBS starts the feature search from the previous stopping position. Upon reaching the end of the feature index array, the search is restarted from the beginning.

#### Boosting

In addition to SFS and the modified SBS, we also use boosting which has been shown to perform very well on the PC-IMAC-Cu data set in [22]. To determine the merit of this *embedded *feature selection approach, we created two versions of the boosting algorithm. The first version is a standard boosting algorithm [23] that uses a weighted nearest centroid method as the base learner. As in the standard nearest centroid, the first round of boosting assigns equal weights to each sample and calculates the nearest centroid for each of the two classes. Each training sample is then classified and re-weighted based on the outcome of classification. If a sample is misclassified, it receives a higher weight (for the next boosting round), whereas if the sample was correctly classified its weight is decreased. The next round of boosting creates new centroids based on the adjusted sample weights and the process repeats itself until training error becomes zero or a predefined number of boosting rounds is reached. This version of the algorithm does not perform feature selection and is used to assess the performance of the second version of boosted nearest centroid algorithm.

The second version of the algorithm extends the boosting algorithm by enabling feature selection. This version, called boosted feature extraction (boostedFE), is similar to sequential forward selection (SFS) in that during each round of boosting the algorithm searches over all features and selects a single best feature upon which to build the weighted nearest centroid classifier. Although variants of this approach have been used in [22] and [27], to the best of our knowledge this is the first time the boostedFE algorithm has been coupled with the (weighted) nearest centroid classifier. The finer aspects of this algorithm are presented in the discussion section of this paper.

### Dimensionality reduction

Feature selection algorithms attempt to select relevant features with respect to the performance task, or conversely remove redundant or irrelevant ones. In contrast, the goal of dimensionality reduction techniques is to literally transform the raw input features while preserving the global information content. In essence, the dimensionality reduction algorithms attempt to extract features capable of *reconstructing *the original high dimensional data, irrespective to the classification label assigned to each data point. For example, principle components analysis (PCA) [14] attempts to find a linear combination of principal components that preserves the variance of the data. In order to test dimensionality reduction algorithms, we have procured the Q5 code used in [17], which uses PCA in conjunction with linear discriminant analysis (LDA) to classify the sample mass spectra. Briefly, PCA projects the MS spectra onto a low dimensional linear manifold required by the LDA algorithm, which cannot use more features than training instances. In turn the LDA algorithm attempts to project the data onto a hyperplane which minimizes within-class scatter, while maximizing between-class distance. Once the data has been projected into the LDA subspace, the nearest centroid approach is used to classify new instances. In our experiments, we test both PCA/LDA + nearest centroid as well as PCA + nearest centroid approaches. This design is meant to assess the merit of individual components, namely PCA and LDA.

### Empirical evaluation

We conducted experiments on three ovarian and two prostate data sets, previously used in [1,2,4,17,19,20,22]. Sets OC-H4, OC-WCX2a, OC-WCX2b, and PC-H4 contain 15,156 features (i.e., m/z values), while the last data set PC-IMAC-Cu contains 16,382 features.

**Figure 1:**
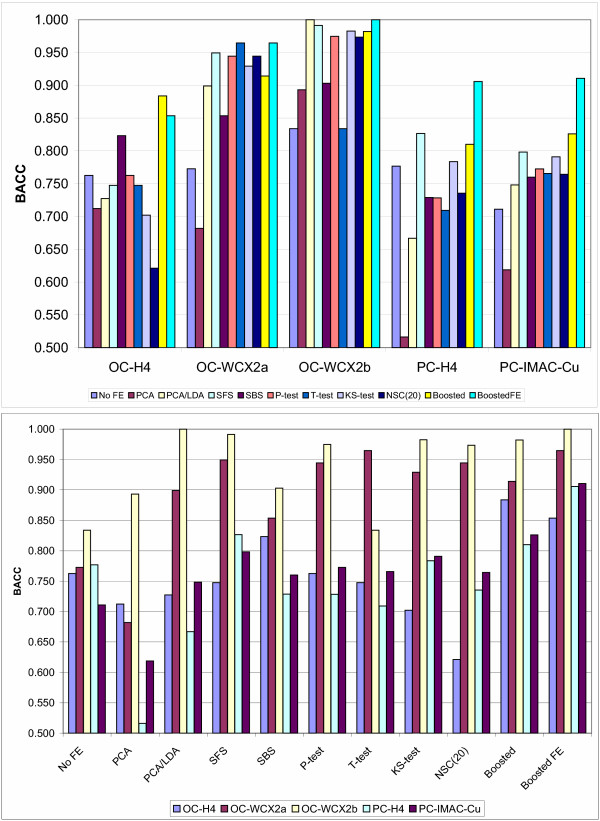
**Performance of Feature Extraction Algorithms on five cancer data sets**. Both graphs show balanced accuracy (BACC) score. **Top: **Results grouped by data set. **Bottom: **Results grouped by feature extraction algorithm.

We used a stratified three-fold cross-validation procedure, for all experiments, whereby each data set was split into three subsets of equal size. Each test fold used one of the three subsets with the remaining two subsets used for training. Within the training phase an internal leave-one-out cross-validation (LOOCV) loop was used for for all feature selection methods (with the exception of dimensionality reduction approaches). In this manner, test set performance remains unbiased by the feature selection process. For PCA and PCA/LDA algorithms, the maximal number of principal components usable by the LDA algorithm was selected and is further described in [17]. The results presented in Figure 1 and Tables 1, 2, 3, 4, 5 express performance statistics averaged over the three *test *folds. Balanced accuracy (BACC) is taken as the arithmetic mean of sensitivity and specificity and is formally defined in the List of Abbreviations section along with the rest of the performance measures. The BACC measure is related to the standard *BER *(Balanced Error Rate), where *BER *= 1 - *BACC *is commonly used for evaluation of feature selection algorithms [16].

### Classification accuracy

Figure 1 and Table 3 present balanced accuracy (BACC) results across the five data sets. The results indicate that boosting based feature extraction (boostedFE) produces the most significant improvement in classification accuracy with respect to performance of the nearest centroid algorithm without feature selection (NoFE). On four of the five data sets boostedFE attained equal or better BACC than any other algorithm tested. On the OC-H4 data set boosting without feature selection slightly outperformed boostedFE algorithm by approximately 3%. However, the difference is not statistically significant as indicated by the paired student-t test at 95% significance level (the probability of the null hypothesis being true is 73.7%). In all other cases boostedFE outperformed the other algorithms including the boosted nearest centroid algorithm.

To make our results comparable with those of Qu et al. in [22], we reran the boosted feature extraction algorithm using ten fold cross-validation scheme on the PC-IMAC-Cu data set and obtained BACC of 98.1%. More specifically our algorithm attained 100% specificity and 96.25% sensitivity. Qu et al. achieved a 98.5% sensitivity and 97.9% specificity averaged over ten 90/10% randomized train/test splits. However, their boosted decision stumps algorithm required 500 rounds of boosting to achieve such a high performance level. As a result, identification of relevant features and their significance is made difficult if not impossible. To find at least some of the relevant features within the PC-IMAC-Cu data set, in [22] the researchers employed the BDSFS algorithm which found 21 relevant features but had a significantly lower accuracy. In contrast, our boostedFE nearest centroid algorithm only required, on average, 5(± 2.8) boosting rounds to achieve comparable classification accuracy. To be fair, we note that the the BDS and BDSFS algorithms used in [22] were ran on pre-processed data, whereby 124 peaks were extracted by the AUC procedure. Hence the performance of our boostedFE algorithm is only comparable in terms of classification accuracy and the number of features selected to the BDS + AUC preprocessing. The quality of features in terms of biological relevance cannot be assessed using this or any of the other tested datasets due to i) biologically confounding factors introduced during sample acquisition and ii) ill-defined data preprocessing steps (the next section discusses these topics in more detail).

The rest of the tested algorithms did not produce consistent results. Some algorithms performed well on one or two of the data sets, but not on all of them as shown in Table 3 (and Figure 1, bottom graph). In contrast, boostedFE consistently produced high quality results on all the tested data sets. In addition, boostedFE produced results with the lowest variance across the cross-validation folds as shown in Tables 1 and 2 by low standard deviation scores. Again, the OH-H4 data set is the exception, where boostedFE has a high standard deviation for the BACC score. A closer look at Table 1 shows that the boostedFE algorithm had 100% (± 0.0) sensitivity but only 70.7% (± 0.26) specificity.

In terms of merely increasing the classification accuracy without performing feature selection, the standard boosting algorithm improved average performance by over 11% as seen in Table 3. Analysis of the training data revealed that in most cases boosting terminated in less than 21 rounds, indicating that for the five datasets used in this study, very few prototypes were needed for accurate sample classification. To see this, recall that in each round of boosting two centroids are produced, one for each class but the size of the training set ranges from 130 samples to 212 samples. Hence, boosting effectively abstracted the training samples into prototypes, producing about 21 class prototypes for each class. Unfortunately, this approach is unlikely to provide insight into the underlying biological factors, provided they exist, due to its use of the full mass spectra.

Surprisingly, the sequential backward selection (SBS) performed rather poorly across all relevant aspects, such as accuracy, running times and size of selected feature subsets. Even more surprising was the poor classification accuracy of T-test, NSC(20), and PCA/LDA algorithms, which appear highly accurate in publications [26] and [17]. Again, the effects of pre-processing steps need to be factored in when comparing our results and those of other studies. Detailed experimental results of this study are presented in Tables 1 and 2 in order to show additional performance statistics such as sensitivity, specificity and positive predictive value obtained under our experimental conditions.

For the OC-H4 data set, it appears that the filter-based methods, SFS, and NSC(20) improve specificity at the cost of decreased sensitivity. In contrast, SBS and boosting based methods improve both with respect to the basic nearest centroid algorithm. This trend resurfaces again for the PC-H4 dataset. This time all algorithms increase specificity at the cost of decreased sensitivity. It is interesting to note that both methods were created via the Ciphergen H4 ProteinChip array and both datasets had their baseline subtracted.

### Feature sets

Table 4 presents statistics on sizes of selected feature subsets. Clearly, the SBS algorithm produces the largest subsets, while the rest of the algorithms produce feature subsets of significantly smaller size. In contrast, the SFS and T-test consistently select very small sets of features across data sets. The boostedFE algorithm also performs quite consistently in terms of the number of features selected. For PCA and PCA/LDA there is no clear way to identify relevant features. However, the number of principal components can be viewed as the degree of compression for a given data set. The number of components for PCA and PCA/LDA is the same and, furthermore, it is constant for a given data set since we select the maximal number of principal components (as in [17]) usable by LDA. In turn, the number of usable dimensions for LDA is given by: *min*(#*samples*, #*features*) – #*classes *and ranges between 130 and 212 dimensions for our data sets. Hence the number of dimensions used by PCA and PCA/LDA algorithms is comparable to the size of the feature sets selected by the SBS algorithm.

### Computational cost comparison

Table 5 shows the computational costs of each feature selection algorithm run on each of the data sets. All experiments were conducted on a dual CPU Athlon 1400+, running Linux. The algorithms were implemented in Matlab 6.5. As expected, filters and dimensionality reduction algorithms have low computational costs. This is due to the fact that the computational complexity of these algorithms is largely governed by sample size. Hence, the run-time performance reflects the small sample size, as compared to the number of features, within the tested data sets. On the other hand, wrapper-based and boosting approaches are computationally much more expensive, in some cases by several orders of magnitude. This is due to fact that feature subsets are evaluated by training a classifier and evaluating performance on a validation set. In our case we use LOOCV, an even costlier but more accurate approach for evaluating the quality of a set of features. However, since the LOOCV approach was also used for the filter methods, the added computational costs can be directly attributed to repeated classifier training. The nearest shrunken centroid method has an additional factor influencing computational cost, namely the number of △ values examined. The NSC(20) used only 20. We also tested NSC(200) which attained very similar classification results at the cost exceeding that of SBS.

## Discussion

While it was expected that SBS would be the most costly algorithm, and that it would produce the largest feature subsets, what is surprising is the noticeably poor overall performance as seen from Table 3. It appears that the additional heuristics we have added to make the algorithm tractable, had a negative impact on the performance of SBS, or that it is simply a poor choice for feature selection in the presence of so many features. On the other hand, SFS is computationally nearly an order of magnitude cheaper than SBS, produces compact feature sets, and has the second best balanced accuracy after boostedFE. From the filter-based approaches, both the KS-test and P-test outperform the T-test in terms of both classification accuracy and running times. T-test, on the other hand, consistently produces very stable features sets as seen from Table 4. Out of the three filter approaches tested, only the T-test appears in the surveyed literature. The P-test, has been used in [11] for gene selection in DNA microarrays. To the best of our knowledge we are the first to use the Kolmogorov-Smirnov test for feature selection in proteomics.

Unexpectedly, the subspace projection methods, namely PCA and PCA/LDA do not perform well under the outlined experimental conditions. This is clearly in contradiction to the results presented in [17]. In fact, Table 3 shows that the nearest centroid classifier without feature selection outperforms PCA on all but one data set. Intuitively, the poor performance of PCA, causes the PCA/LDA combination to also perform rather poorly on three of the five data sets. We should note that the randomized re-sampling testing strategy as used in [17] and [22] along with a number of other papers has been shown to be overly optimistic due to the correlations between test and train sets (see [7] and references within for a detailed explanation). Hence, we believe that this testing methodology has a significant impact on performance. On the other hand, stratified cross-validation approaches, such as the one we have adopted in this paper, remove correlations between test sets, giving more accurate performance estimates. As a consequence, all performance statistics appear 'deflated' in comparison to results reported in previous studies. However, we believe that these, 3-fold cross-validation results, provide more realistic performance estimates and can be used to make statistically sound inferences.

### Nearest centroid, SFS, and boosting

The choice of nearest centroid classifier to study feature selection was not an arbitrary one. Although the nearest centroid is one of the simplest classifiers found in the literature, nevertheless it is capable of classifying raw mass spectra without any feature selection. In addition, it is extremely fast and therefore allows the use of costly wrapper methods, such as SFS, SBS, and boostedFE, which may otherwise be intractable. Hence, not only does the nearest centroid classifier able to provide a base-line for evaluation of feature selection algorithms, it also allows us to test a number of algorithms previously inapplicable in the domain of proteomic mass spectrometry. For the two class problems considered, the nearest centroid algorithm is linear and implicitly encodes a thresholding hyperplane separating the two classes. However, when combined with boosting the algorithm becomes capable of encoding non-linear boundaries. As mentioned previously, the use of boosting effectively abstracts the training samples into prototypes. Integration of sequential forward selection (SFS) yields a further improvement. By merging weighted nearest centroid with boosting and SFS, the new algorithm is able to simultaneously select relevant features and learn a highly accurate classifier. Thus boostedFE, fulfills both rolls as a feature selection and classification algorithm. By testing the nearest centroid without feature selection, SFS, boosting, and boostedFE, we can easily gauge the effect each component has on the performance of boostedFE. In fact this piece-wise analysis can easily explain why boosting outperformed boosting FE on the OC-H4 data set.

From Table 1, we can see that SFS performed worse than NoFE (meaning nearest centroid without feature selection), hence when boosting and SFS were used together the net effect actually lowered performance in comparison to boosting without feature selection. More specifically, we can see from Table 1 that the specificity of SFS for the OC-H4 data set was extremely low (51.5%) and was accompanied with a very high standard deviation of (± 42%).

### Feature analysis

The aim of this paper was to profile a number of feature selection algorithms coupled with the nearest centroid classifier. Our goal was to examine performance in terms of computational time, feature set sizes and, most importantly, classification accuracy. However, due to the concerns raised in [2,24] regarding the quality of ovarian and prostate cancer data, we make no attempt to interpret the results of feature selection from a biological standpoint. Furthermore, data preprocessing strategies, themselves being actively studied [3], should also be examined in future investigations due to their influence on feature selection and classification results. In order to truly assess biological underpinnings of discriminative m/z values, it is imperative that datasets free from flaws, which confound biology with instrument noise, collection bias, and/or other "artifacts of sample effects" [2], are used in further studies. In addition, the effectiveness of preprocessing methods can only be assessed with respect their ability to improve identification of relevant biological factors governing class discrimination.

## Conclusion

Mass spectrometry based disease diagnosis is an emerging field, with the potential to revolutionize early medical diagnosis. However, due to the vast amount of information captured by the high-resolution mass spectrometry techniques, the supervised training of classifiers is problematic. Specifically, the many thousands of raw attributes forming the mass spectra frequently contain a large amount of redundancy, information irrelevant to a particular disease, and measurement noise. Therefore, aggressive feature selection techniques are crucial for learning high-accuracy classifiers and realizing the full potential of mass spectrometry based disease diagnosis. This paper analyzed dimensionality reduction, filter, wrapper, and boosting based approaches to feature selection and compared the results to previously published state-of-the-art performance. In addition, a novel combination of nearest centroid classifier coupled with boosting based feature selection (boostedFE) was presented and evaluated. Experimental results indicate that sequential forward selection, P-test, and KS-test perform reasonably well across the proteomic data sets we acquired. However, the aforementioned algorithms lack consistency. On the other hand, the proposed boostedFE algorithm greatly reduces the dimensionality of the data and *significantly *improves classification accuracy. In contrast to all other algorithms, its performance is much more consistent across all five data sets used in the experiments.

Future research will investigate the extent to which the features selected by the boostedFE approach can be used in conjunction with more sophisticated classifiers, such as artificial neural networks and support vector machines. In addition, future studies should investigate whether the boostedFE + nearest centroid combination can serve as a meta-wrapper for more sophisticated classification algorithms. From a biological perspective, the significance of the selected features and their value in identifying potential biomarkers should be investigated. A prerequisite for this task is the production of datasets where biological factors are not confounded by instrumentation noise, sample acquisition bias and/or other experimental design flaws. The production of these datasets would also enable future studies to accurately assess the effectiveness of preprocessing techniques, critical for producing diagnostic tools which indeed base classification on underlying biological factors encoded within the mass spectra.

## List of abbreviations

In this section we define the various measures used. Respectively, *TP*, *TN*, *FP*, *FN*, stand for the number of true positive, true negative, false positive, false negative samples at classification time.

**Sensitivity **is defined as  and is also known as Recall.

**Specificity **is defined as .

**PPV (Positive Predictive Value) **is defined as  and is also known as Precision.

**NPV (Negative Predictive Value) **is defined as .

**BACC (Balanced Accuracy) **is defined as  This measure defines the average of sensitivity and specificity.

**% correct **is defined as  and measures the overall percentage of samples correctly classified.

## Figures and Tables

**Table 1:** Detailed performance statistics for ovarian cancer data sets Bold columns represent the mean of the respective performance measure, while columns labeled as (std) correspond to the standard deviation across the three cross-validation folds.

***OC-H4***	**Corr**	Corr(std)	**BACC**	3ACC(std	**Spec**	Spec(std)	**Sens**	Sens(std)	**PPV**	PPV(std)
No FE	**0.763**	0.05	**0.763**	0.05	**0.848**	0.16	**0.677**	0.11	**0.841**	0.13
PCA	**0.712**	0.07	**0.712**	0.07	**0.727**	0.25	**0.697**	0.12	**0.768**	0.20
PCA/LDA	**0.727**	0.07	**0.727**	0.07	**0.636**	0.31	**0.818**	0.18	**0.744**	0.19
SFS	**0.747**	0.22	**0.747**	0.22	**0.980**	0.02	**0.515**	0.42	**0.931**	0.06
SBS	**0.823**	0.08	**0.823**	0.08	**0.899**	0.13	**0.747**	0.08	**0.891**	0.12
P-test	**0.763**	0.20	**0.763**	0.20	**0.929**	0.05	**0.596**	0.38	**0.863**	0.09
T-test	**0.747**	0.19	**0.747**	0.19	**0.929**	0.02	**0.566**	0.38	**0.856**	0.08
KS-test	**0.702**	0.22	**0.702**	0.22	**0.909**	0.09	**0.495**	0.35	**0.766**	0.28
NSC(20)	**0.621**	0.19	**0.621**	0.19	**0.949**	0.06	**0.293**	0.32	**0.743**	0.29
Boosted	**0.884**	0.06	**0.884**	0.06	**0.990**	0.02	**0.778**	0.11	**0.986**	0.03
Boosted FE	**0.854**	0.13	**0.854**	0.13	**1.000**	0.00	**0.707**	0.26	**1.000**	0.00

***OC-WCX2a***	**Corr**	Corr(std)	**BACC**	3ACC(std	**Spec**	Spec(std)	**Sens**	Sens(std)	**PPV**	PPV(std)

No FE	**0.773**	0.09	**0.773**	0.09	**0.828**	0.02	**0.717**	0.18	**0.800**	0.05
PCA	**0.682**	0.18	**0.682**	0.18	**0.687**	0.14	**0.677**	0.25	**0.671**	0.18
PCA/LDA	**0.899**	0.02	**0.899**	0.02	**0.889**	0.10	**0.909**	0.06	**0.900**	0.09
SFS	**0.949**	0.03	**0.949**	0.03	**0.980**	0.03	**0.919**	0.05	**0.979**	0.04
SBS	**0.854**	0.15	**0.854**	0.15	**0.929**	0.08	**0.778**	0.23	**0.903**	0.12
P-test	**0.944**	0.03	**0.944**	0.03	**0.970**	0.03	**0.919**	0.06	**0.969**	0.03
T-test	**0.965**	0.02	**0.965**	0.02	**0.949**	0.05	**0.980**	0.02	**0.953**	0.04
KS-test	**0.929**	0.02	**0.929**	0.02	**0.970**	0.03	**0.889**	0.05	**0.968**	0.03
NSC(20)	**0.944**	0.04	**0.944**	0.04	**0.990**	0.02	**0.899**	0.08	**0.989**	0.02
Boosted	**0.914**	0.06	**0.914**	0.06	**1.000**	0.00	**0.828**	0.12	**1.000**	0.00
Boosted FE	**0.965**	0.01	**0.965**	0.01	**1.000**	0.00	**0.929**	0.02	**1.000**	0.00

***OC-WCX2b***	**Corr**	Corr(std)	**BACC**	3ACC(std	**Spec**	Spec(std)	**Sens**	Sens(std)	**PPV**	PPV(std)

No FE	**0.837**	0.14	**0.834**	0.12	**0.822**	0.07	**0.846**	0.20	**0.891**	0.05
PCA	**0.901**	0.05	**0.893**	0.03	**0.867**	0.03	**0.920**	0.07	**0.926**	0.02
PCA/LDA	**1.000**	0.00	**1.000**	0.00	**1.000**	0.00	**1.000**	0.00	**1.000**	0.00
SFS	**0.992**	0.01	**0.991**	0.01	**0.989**	0.02	**0.994**	0.01	**0.994**	0.01
SBS	**0.901**	0.14	**0.903**	0.13	**0.911**	0.10	**0.895**	0.17	**0.942**	0.07
P-test	**0.980**	0.02	**0.975**	0.03	**0.956**	0.05	**0.994**	0.01	**0.976**	0.03
T-test	**0.837**	0.07	**0.834**	0.04	**0.822**	0.05	**0.846**	0.13	**0.897**	0.01
KS-test	**0.984**	0.02	**0.983**	0.02	**0.978**	0.04	**0.988**	0.01	**0.988**	0.02
NSC(20)	**0.972**	0.02	**0.973**	0.03	**0.978**	0.04	**0.969**	0.03	**0.988**	0.02
Boosted	**0.980**	0.01	**0.982**	0.00	**0.989**	0.02	**0.975**	0.02	**0.994**	0.01
Boosted FE	**1.000**	0.00	**1.000**	0.00	**1.000**	0.00	**1.000**	0.00	**1.000**	0.00

**Table 2:** Detailed performance statistics for prostate cancer data sets Bold columns represent the mean of the respective performance measure, while columns labeled as (std) correspond to the standard deviation across the three cross-validation folds.

***PC-H4***	**Corr**	Corr(std)	**BACC**	3ACC(std	**Spec**	Spec(std)	**Sens**	Sens(std)	**PPV**	PPV(std)
No FE	**0.732**	0.05	**0.777**	0.06	**0.698**	0.05	**0.855**	0.09	**0.439**	0.06
PCA	**0.530**	0.20	**0.516**	0.18	**0.540**	0.24	**0.493**	0.21	**0.248**	0.11
PCA/LDA	**0.692**	0.15	**0.667**	0.14	**0.710**	0.22	**0.623**	0.33	**0.431**	0.17
SFS	**0.885**	0.05	**0.827**	0.17	**0.929**	0.03	**0.725**	0.36	**0.728**	0.03
SBS	**0.773**	0.03	**0.729**	0.09	**0.806**	0.11	**0.652**	0.27	**0.498**	0.07
P-test	**0.813**	0.02	**0.728**	0.11	**0.877**	0.08	**0.580**	0.28	**0.572**	0.07
T-test	**0.816**	0.04	**0.709**	0.14	**0.897**	0.05	**0.522**	0.31	**0.575**	0.07
KS-test	**0.826**	0.04	**0.784**	0.14	**0.857**	0.08	**0.710**	0.35	**0.579**	0.05
NSC(20)	**0.791**	0.04	**0.736**	0.10	**0.833**	0.12	**0.638**	0.31	**0.529**	0.07
Boosted	**0.850**	0.06	**0.810**	0.11	**0.881**	0.04	**0.739**	0.22	**0.627**	0.10
Boosted FE	**0.960**	0.01	**0.906**	0.03	**1.000**	0.00	**0.812**	0.07	**1.000**	0.00

***PC-IMAC-Cu***	**Corr**	Corr(std)	**BACC**	3ACC(std	**Spec**	Spec(std)	**Sens**	Sens(std)	**PPV**	PPV(std)

No FE	**0.709**	0.13	**0.711**	0.13	**0.767**	0.14	**0.655**	0.12	**0.750**	0.15
PCA	**0.618**	0.07	**0.619**	0.07	**0.654**	0.21	**0.583**	0.20	**0.652**	0.08
PCA/LDA	**0.746**	0.03	**0.748**	0.03	**0.818**	0.07	**0.679**	0.04	**0.800**	0.06
SFS	**0.795**	0.03	**0.798**	0.03	**0.912**	0.14	**0.685**	0.07	**0.914**	0.13
SBS	**0.758**	0.15	**0.760**	0.15	**0.818**	0.15	**0.702**	0.15	**0.802**	0.17
P-test	**0.771**	0.06	**0.773**	0.06	**0.843**	0.15	**0.702**	0.05	**0.840**	0.12
T-test	**0.765**	0.06	**0.766**	0.06	**0.805**	0.10	**0.726**	0.09	**0.803**	0.08
KS-test	**0.789**	0.03	**0.791**	0.03	**0.862**	0.09	**0.720**	0.05	**0.854**	0.07
NSC(20)	**0.761**	0.09	**0.764**	0.09	**0.868**	0.12	**0.661**	0.17	**0.849**	0.10
Boosted	**0.823**	0.05	**0.826**	0.05	**0.950**	0.09	**0.702**	0.11	**0.949**	0.09
Boosted FE	**0.908**	0.02	**0.911**	0.02	**1.000**	0.00	**0.821**	0.03	**1.000**	0.00

**Table 3:** Overall performance comparison Performance of each feature extraction algorithms averaged across data sets. Balanced accuracy (BACC) reported in increasing order.

	Average BACC	*(+/-)*	OC-H4	OC-WCX2a	OC-WCX2b	PC-H4	PC-IMAC-Cu
**PCA**	**0.684**	*0.139*	0.712	0.682	0.893	0.516	0.619
**No FE**	**0.771**	*0.044*	0.763	0.773	0.834	0.777	0.711
**T-test**	**0.804**	*0.100*	0.747	0.965	0.834	0.709	0.766
**NSC(20)**	**0.808**	*0.148*	0.621	0.944	0.973	0.736	0.764
**PCA/LDA**	**0.808**	*0.137*	0.727	0.899	1.000	0.667	0.748
**SBS**	**0.814**	*0.070*	0.823	0.854	0.903	0.729	0.760
**P-test**	**0.837**	*0.114*	0.763	0.944	0.975	0.728	0.773
**KS-test**	**0.838**	*0.115*	0.702	0.929	0.983	0.784	0.791
**SFS**	**0.863**	*0.103*	0.747	0.949	0.991	0.827	0.798
**Boosted**	**0.883**	*0.070*	0.884	0.914	0.982	0.810	0.826
**Boosted FE**	**0.927**	*0.057*	0.854	0.965	1.000	0.906	0.911

**Table 4:** Feature set size comparison

	OC-H4	(+/-)	OC-WCX2a	(+/-)	OC-WCX2b	(+/-)	PC-H4	(+/-)	PC-IMAC	(+/-)
**SFS**	**3**	1.15	**3**	1.15	**3**	1.15	**4**	1.00	**5**	1.53
**SBS**	**139**	195.19	**94**	120.75	**454**	769.62	**205**	162.69	**136**	144.24
**P-test**	**2**	1.53	**6**	3.06	**41**	66.97	**3**	2.08	**1**	0.58
**T-test**	**5**	2.08	**3**	0.58	**2**	0.58	**2**	0.58	**3**	0.58
**KS-test**	**2**	1.00	**7**	4.36	**63**	106.52	**2**	1.53	**2**	0.58
**Boosted FE**	**7**	3.51	**3**	0.58	**3**	0.58	**8**	1.15	**10**	5.03

**Table 5:** Computational cost comparison Results presented in CPU seconds and in increasing order. All experiments were conducted using Matlab code on a dual CPU Athlon 1400+ running Linux.

	Ave. CPU Time	*(+/-)*	OC-H4	OC-WCX2a	OC-WCX2b	PC-H4	PC-IMAC-Cu
**No FE**	**1.19**	*0.40*	0.84	0.87	1.11	1.31	1.82
**P-test**	**2.42**	*1.11*	1.41	1.58	4.12	2.08	2.90
**PCA**	**19.39**	*7.70*	12.69	12.08	17.41	25.57	29.21
**PCA/LDA**	**20.58**	*8.08*	13.53	12.95	18.52	26.88	31.03
**KS-test**	**27.36**	*3.07*	25.56	24.55	29.96	25.40	31.34
**Boosted**	**543.42**	*332.13*	371.73	134.56	507.62	688.22	1014.97
**T-test**	**649.84**	*39.39*	622.97	623.37	645.66	639.14	718.04
**SFS**	**3164.70**	*1477.13*	2178.24	2175.75	2516.42	3269.37	5683.70
**BoostedFE**	**3356.89**	*2236.51*	2679.97	1336.25	1841.57	3997.65	6928.99
**NSC(20)**	**5434.20**	*3321.34*	3717.30	1345.60	5076.20	6882.20	10149.70
**SBS**	**23934.82**	*6655.33*	17032.94	17244.80	29913.61	24574.69	30908.07
